# A 68-year old male presenting with rhabdomyolysis-associated acute kidney injury following concomitant use of elvitegravir/cobicistat/emtricitabine/tenofovir disoproxil fumarate and pravastatin/fenofibrate: a case report

**DOI:** 10.1186/s13256-015-0671-z

**Published:** 2015-09-08

**Authors:** Veronique Suttels, Eric Florence, John Leys, Marc Vekemans, Jef Van den Ende, Erika Vlieghe, Chris Kenyon

**Affiliations:** 1 Tropical Disease Unit, Antwerp University Hospital, Wilrijkstraat 10, Edegem, Belgium; 2 Department of Clinical Sciences, Institute of Tropical Medicine, Kronenburgstraat 43/3, 2000 Antwerp, Belgium; 3 Department of Pharmacy, Antwerp University Hospital, Wilrijkstraat 10, Edegem, Belgium

## Abstract

**Introduction:**

We present what we believe to be the first case in the literature of rhabdomyolysis-induced renal failure caused by a probable drug interaction between elvitegravir/cobicistat/emtricitabine/tenofovir disoproxil fumarate (EVG/COBI/FTC/TDF) and pravastatin/fenofibrate.

**Case presentation:**

A 68-year old Caucasian man presented with progressive pain in both legs two weeks after commencing treatment with EVG/COBI/FTC/TDF. He was found to have biochemical evidence of rhabdomyolysis and acute renal failure.

**Conclusion:**

We emphasize the need for post marketing surveillance of adverse effects of new products. Pharmacokinetic studies are necessary to investigate the levels of pravastatin in patients taking COBI and fenofibrate with and without other comorbidities. Meanwhile, we suggest that creatine kinase levels should be monitored and patients advised to report myalgias when using concomitant EVG/COBI/FTC/TDF and pravastatin/fenofibrate. This case serves as an important reminder to use estimated glomerular filtration rates rather than serum creatinine levels when choosing new medications. If potentially nephrotoxic combinations are started in patients with borderline estimated glomerular filtration rates, it may be prudent to check these filtration rates more frequently than usual. In patients with reduced estimated glomerular filtration rates, potentially nephrotoxic combinations should be avoided wherever possible.

## Introduction

The novel single tablet regimen of elvitegravir (EVG), cobicistat (COBI), emtricitabine (FTC), and tenofovir disoproxil fumarate (TDF) is used to treat human immunodeficiency virus (HIV)-1 infection. Contemporary guidelines do not advise caution, dose adjustments, or enhanced monitoring of patients who are taking concomitant pravastatin and EVG/COBI/FTC/TDF [1]. We describe the case of a 68-year-old patient who developed rhabdomyolysis and acute kidney injury (AKI) following the commencement of EVG/COBI/FTC/TDF. To the best of our knowledge, this is the first such case reported in the literature.

## Case presentation

Our patient, a 68-year old Caucasian man, was first diagnosed with HIV-1 infection in 1993. After six years on FTC, zidovudine, and ritonavir-boosted lopinavir he was switched to EVG/COBI/FTC/TDF owing to the development of dyslipidemia and a desire for treatment simplification. He also had hypertension, gout, and impaired renal function. His weight was 73kg with a body mass index of 24.7kg/m^2^. At the time of the therapy switch, his serum creatinine level was in the normal range (1.16mg/dL) and results from a urine analysis were normal, but his estimated glomerular filtration rate (eGFR) via both the Modification of Diet in Renal Disease (MDRD) and the Cockcroft–Gault formulas was slightly reduced (66.6mL/min per 1.73m^2^ and 62.9mL/min per 1.73m^2^, respectively). Other medications he continued to take were acetyl salicylic acid 80mg, lisinopril 5mg, pravastatin 40mg, fenofibrate 267mg, and allopurinol 100mg (all once daily). Our patient’s age, comorbidities, polypharmacy, and slightly impaired renal function were possible risk factors for altered drug effects and interactions [2].

Two weeks after the switch, our patient developed progressive pain in both calves and thighs. The following week he experienced anorexia, nausea, and dark urine, and presented to our hospital with clinical and biochemical evidence of established rhabdomyolysis and acute tubular necrosis.

A laboratory investigation on admission revealed the following: creatine kinase (CK) >20,000U/L, creatinine 11.9mg/dL, urea 248mg/dL, and eGFR 4mL/min per 1.73m^2^. His electrolytes and thyroid function tests were normal. A urine analysis showed evidence of acute tubular necrosis. He tested negative for the rs4363657 single-nucleotide polymorphism located within *SLCO1B1* gene. This gene encodes the organic anion transporting polypeptide 1B1 (OATP1B1) that has been associated with statin-induced myopathy defined as muscle pain or weakness with elevated CK levels [3].

All medication was discontinued and intravenous fluid replacement was initiated. Gradually, the myalgia resolved and his renal function recuperated without the need for dialysis. Antiretroviral therapy was recommenced three weeks after admission, first with darunavir, norvir, lamivudine, and abacavir, adjusted according to his eGFR. After one week, the darunavir was changed to lopinavir because of persisting gastrointestinal complaints. On discharge, four weeks after admission, his serum creatinine was 4.8mg/dL and his eGFR was 12mL/min per 1.73m^2^. At last evaluation five months post-discharge, he was asymptomatic and his kidney function showed further improvement (serum creatinine of 1.51mg/dL and eGFR of 47mL/min per 1.73m^2^). His CD4 lymphocyte count was 810 cells/μL and he had an undetectable viral load (<20 copies/mL).

## Discussion

**Fig. 1:**
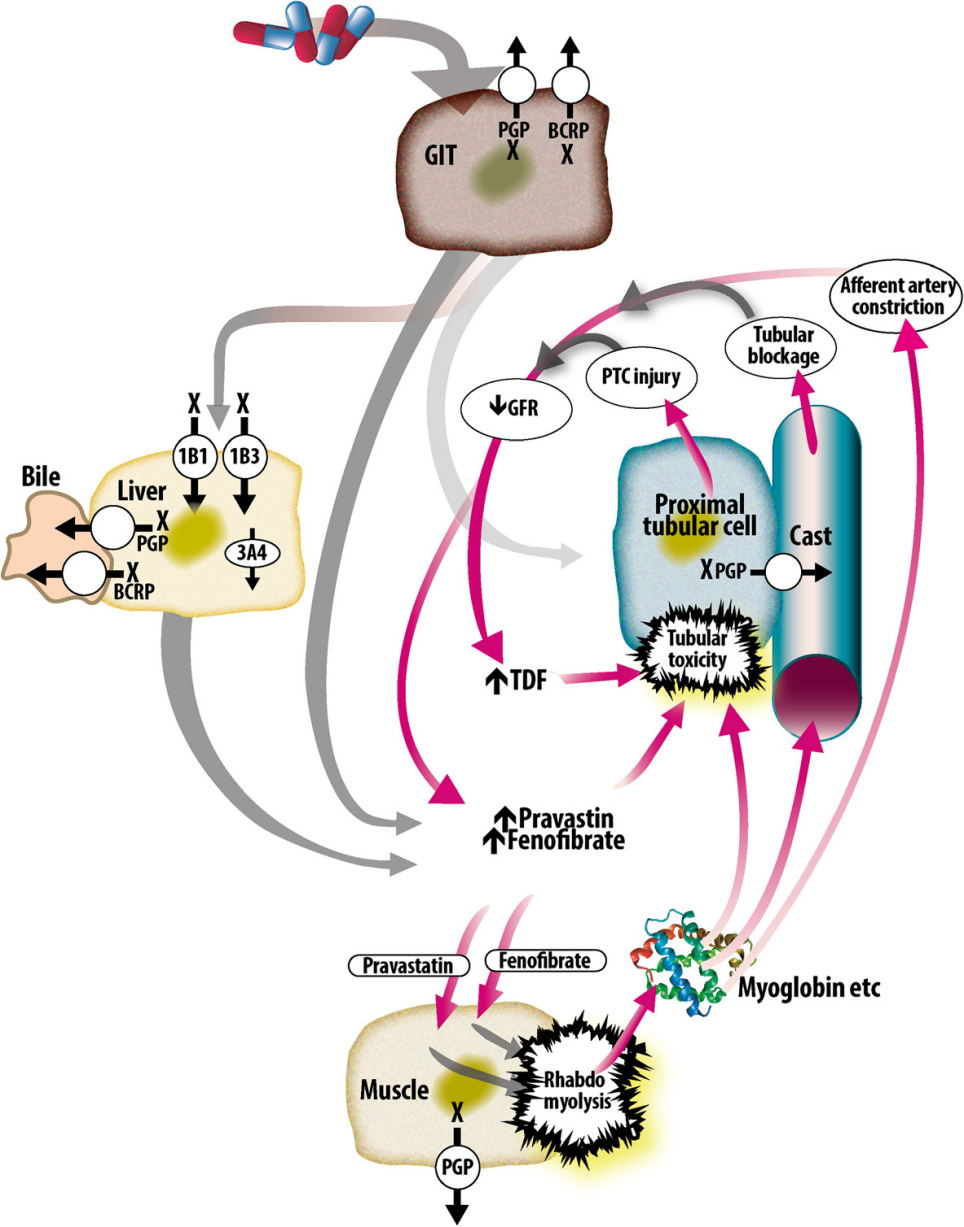
Possible pathophysiological mechanisms leading to rhabdomyolysis and acute kidney injury in this case. *1B1* and *1B3* organic anion transporters 1B1 and 1B3, *3A4* cytochrome P450 3A4, *BCRP* breast cancer resistance protein, *GIT* gastrointestinal tract, *PGP* P-glycoprotein, *PTC* proximal tubular cell, *X* inhibited by cobicistat

The nature and timing of our patient’s presentation with rhabdomyolysis-induced AKI two weeks after switching to EVG/COBI/FTC/TDF suggests a drug-induced etiology. His Naranjo Adverse Drug Reaction Probability Score was 6 (indicating a probable adverse drug event) [4]. It is possible that a number of factors combined to cause the rhabdomyolysis (Fig. 1). His age and reduced eGFR at baseline would have predisposed him to nephrotoxicity from TDF and fenofibrate [5–7]. Besides his chronic HIV infection and hypertension, it is possible that his chronic aspirin treatment, albeit in low doses, may have reduced his baseline renal function [8]. This reduced GFR could have increased his serum levels of fenofibrate, TDF, and, to a lesser extent, pravastatin [2, 6].

COBI is a novel potent CYP3A4 inhibitor, used to boost the levels of EVG in the co-formulation EVG/COBI/FTC/TDF [9]. It also inhibits the secretion of creatinine by the proximal tubule, leading to a slight (<10%) increase in serum creatinine and a decrease of eGFR. There is no evidence of a decline in actual GFR and the decline in eGFR is fully and promptly reversible upon discontinuation [9, 10]. COBI has not been studied in patients with an eGFR under 70mL/min per 1.73m^2^. COBI has a number of other off-target effects. It inhibits CYP2A6, p-glycoprotein (P-gp), and OATP1B1 [11]. Although both pravastatin and rosuvastatin undergo minimal CYP450 metabolism, a pharmacokinetic study showed that COBI increased the maximum serum concentration of rosuvastatin by 89%. This was thought to be due to COBI-induced inhibition of intestinal efflux by breast cancer resistance protein (BCRP) or hepatic uptake by OATPs [12].

Although no pharmacokinetic studies have been performed on the interaction between COBI and pravastatin, COBI would be expected to increase the serum levels of pravastatin by a number of mechanisms: inhibition of BCRP intestinal and biliary efflux pumps; inhibition of the P-gp intestinal, biliary, and renal efflux pumps; and inhibition of OATP1B1-mediated entry into hepatocytes [9, 13]. Pravastatin levels inside skeletal muscle cells may also be increased by COBI-induced inhibition of P-gp efflux from these cells [14]. Statin-induced rhabdomyolysis is thought to result from a number of factors. Drug interactions, particularly those leading to increased levels of statins, have been found to play a prominent role in a number of studies [15–17]. The predicted increase in pravastatin levels in serum and muscle cells in our patient may thus have been sufficient to trigger rhabdomyolysis, which would have caused a further decline in eGFR leading to self-reinforcing increases in TDF and pravastatin/fenofibrate. The observed enhanced risk of rhabdomyolysis when pravastatin is used simultaneously with a high dose fenofibrate may also be mediated by fibrate inhibition of statin excretion and hence higher serum statin levels [18]. It is also possible that EVG played a role. There have been six case reports of raltegravir (another integrase inhibitor) linked to rhabdomyolysis. Pravastatin was used concomitantly in one of these cases [19]. Of potential relevance to our case, this association has only been detected during post marketing surveillance. As a result of these cases, the US Food and Drug Administration now recommends caution in using raltegravir in patients with increased risk for muscle damage [20]. Finally, there have been reports of acute tubular necrosis after adding TDF to lisinopril therapy [21].

## Conclusion

Two large trials reporting the efficacy of EVG/COBI/FTC/TDF were industry sponsored [22, 23]. A Cochrane review found that industry-sponsored clinical trials are significantly more likely than non-commercially funded studies to report favorable efficacy and safety results. Plausible mechanisms for the minimization of side effects include excluding up to 30% due to comorbidities [24].

These considerations justify the need for post marketing surveillance of adverse effects of new products. We believe that pharmacokinetic studies are necessary to investigate the levels of pravastatin in patients on COBI and fenofibrate with and without other comorbidities. Meanwhile, we encourage caution in the concomitant use of EVG/COBI/FTC/TDF and pravastatin/fenofibrate. The case serves as an important reminder to use eGFR rather than serum creatinine when choosing new medications. If potentially nephrotoxic combinations are started in patients with borderline eGFRs, it may be prudent to check the eGFR more frequently than usual. In patients with reduced eGFRs, potentially nephrotoxic combinations should be avoided wherever possible. If there are no alternatives then patients should be closely monitored and advised to report myalgia as promptly as possible.

## Consent

Written informed consent was obtained from the patient for publication of this case report. A copy of the written consent is available for review by the Editor-in-Chief of this journal.

## Abbreviations

AKI: Acute kidney injury
BRCP: Breast cancer resistance protein
CK: Creatine kinase
CYP: Cytochrome P450
eGFR: estimated glomerular filtration rate
EVG/COBI/FTC/TDF: Elvitegravir, cobicistat, emtricitabine, tenofovir disoproxil fumarate
MDRD: Modification of Diet in Renal Disease
OATP: Organic anion transporting polypeptide
P-gp: P-glycoprotein
